# Psychological stress, adverse life events and breast cancer incidence: a cohort investigation in 106,000 women in the United Kingdom

**DOI:** 10.1186/s13058-016-0733-1

**Published:** 2016-07-15

**Authors:** Minouk J. Schoemaker, Michael E. Jones, Lauren B. Wright, James Griffin, Emily McFadden, Alan Ashworth, Anthony J. Swerdlow

**Affiliations:** Division of Genetics and Epidemiology, The Institute of Cancer Research, London, SM2 5NG UK; Division of Breast Cancer Research, The Institute of Cancer Research, London, SW3 6JB UK; Warwick Clinical Trials Unit, the University of Warwick, Warwick, CV4 7AL UK; Nuffield Department of Primary Care Health Sciences, University of Oxford, Oxford, OX2 6GG UK; UCSF Helen Diller Family Comprehensive Cancer Center, San Francisco, CA 94158 USA

**Keywords:** Breast cancer, Bereavement, Cohort studies, Life change events, Stress, Psychological

## Abstract

**Background:**

Women diagnosed with breast cancer frequently attribute their cancer to psychological stress, but scientific evidence is inconclusive. We investigated whether experienced frequency of stress and adverse life events affect subsequent breast cancer risk.

**Methods:**

Breast cancer incidence was analysed with respect to stress variables collected at enrolment in a prospective cohort study of 106,000 women in the United Kingdom, with 1783 incident breast cancer cases. Relative risks (RR) were obtained as hazard ratios using Cox proportional hazards models.

**Results:**

There was no association of breast cancer risk overall with experienced frequency of stress. Risk was reduced for death of a close relative during the 5 years preceding study entry (RR = 0.87, 95 % confidence interval (CI): 0.78–0.97), but not for death of a spouse/partner or close friend, personal illness/injury, or divorce/separation. There was a positive association of divorce with oestrogen-receptor-negative (RR = 1.54, 95 % CI: 1.01–2.34), but not with oestrogen-receptor-positive breast cancer. Risk was raised in women who were under age 20 at the death of their mother (RR = 1.31, 95 % CI: 1.02–1.67), but not of their father, and the effect was attenuated after excluding mothers with breast or ovarian cancer (RR = 1.17, 95 % CI: 0.85–1.61).

**Conclusions:**

This large prospective study did not show consistent evidence for an association of breast cancer risk with perceived stress levels or adverse life events in the preceding 5 years, or loss of parents during childhood and adolescence.

**Electronic supplementary material:**

The online version of this article (doi:10.1186/s13058-016-0733-1) contains supplementary material, which is available to authorized users.

## Background

Breast cancer is the most commonly diagnosed cancer among females in the Western world, and its aetiology entails a multitude of genetic, reproductive, hormonal, and exogenous factors. Women with breast cancer frequently attribute the origin of their breast cancer, however, to psychological factors such as stress [1] although scientific evidence for this is inconclusive.

Stress has been defined theoretically as the response of the body to the presence of external demands or, more subjectively, as the response to the individual’s appraisal of demands depending on their coping abilities [2, 3]. Proposed biological mechanisms for an effect of stress on cancer development include neuroendocrine alterations in the hypothalamus-pituitary-adrenal axis regulating glucocorticoid release and the sympathetic nervous system regulating catecholamine levels [4, 5]. Overactivation of the allostatic system is thought possibly to impair immune response [6] and release of stress hormones has been implicated in DNA repair, tumour cell growth and angiogenesis [7–10]. However, a protective effect of stress on breast cancer risk through suppression of oestrogen secretion has also been proposed [11–13]. Indirect effects of stress on health and cancer risk, through changes in lifestyle and behaviour, are also possible, and stress may render women more susceptible to progression or recurrence of cancer either by affecting recovery or compliance with their treatment.

Past epidemiological studies of exposure to psychological stress and breast cancer risk have predominantly reported breast cancer risk after adverse life events such as bereavement or divorce, as these are relatively easily ascertained, objective measures of major external stressors [14]. Only a few studies have investigated more subjective measures such as perceived experience of stress, which could reflect a subject’s coping in response to stressors. Some have investigated risk in women who were bereaved of their parents during childhood, as the impact of psychological stress might be particularly great during this period of development. Past studies of psychological stress, however, have mainly been case-control or cohort studies with small numbers of cases, or record-linkage-based studies that have had no or limited ability to adjust for confounding variables. They have been methodologically diverse in design, adjustment for confounders, population characteristics and the type of effect measures investigated, and appear to have been subject to publication bias [15]. The studies’ heterogeneous results have been reflected in the contradictory conclusions of reviews [12, 15–18].

To address this issue, large prospective cohort studies are needed because such studies have the substantial advantage over those of retrospective design in that recall bias is avoided. We therefore investigated perceived frequency of stress, experience of adverse life events and bereavement at young ages in relation to breast cancer risk in a large prospective cohort study in the United Kingdom.

## Methods

The Breakthrough Generations Study is a cohort study of over 113,000 women in the UK, aged 16 or older, focused on breast cancer aetiology. The main recruitment phase was during 2003–2010, with baseline recruitment involving a postal questionnaire about established and candidate breast cancer risk factors, and donation of a blood sample. Participants are followed up approximately every 2.5–3 years by postal questionnaires, to obtain updated risk factor information as well as details of breast cancer diagnoses. Full details of the cohort methods have been published previously [19].

Notification of incident cancers is obtained from follow-up questionnaires and ‘flagging’ at the National Health Service Central Registers (NHSCR), virtually complete registers of the population of the country, which notify cancer registrations in subjects ‘flagged’ for the study to authorised medical researchers as well as information on emigrations and deaths. Confirmation of self-reported diagnoses is obtained from the cancer registrations, participants’ general practitioners, pathology reports and other hospital records.

The current analysis is based on all women who joined the Breakthrough Generations Study before 1 July 2012 without previous breast cancer, the recruitment cut-off date reflecting the date by which the first follow-up for the questionnaire was practically complete. Follow-up for breast cancer started at the date of receipt of the recruitment questionnaire and ended on the date of mailing for follow-up questionnaires, or if the follow-up questionnaire was not returned and the woman was covered by ‘flagging’ the earliest of the date the individual’s ‘flagging’, coverage ended (i.e. at death or removal from the NHSCR), or the date after which ‘flagging’ notification is not yet complete (taken to be 1 March 2014). If the follow-up questionnaire was not returned and the woman was not covered by ‘flagging’, the follow-up was truncated at the date of her previous questionnaire.

Data on stress variables were obtained from the baseline questionnaire, which asked whether subjects felt they had been experiencing stress over the last 5 years, to which they were invited to respond ‘never’, ‘occasionally’, ‘frequently’ or ‘continuously’. We combined the ‘never’ and ‘occasionally’ categories in the analyses due to the low number of subjects who reported ‘never’. The questionnaire also asked whether in the last 5 years they had experienced: death of a husband or long-term partner; death of a child, parent or other close relative; death of a close friend; divorce or separation; serious personal illness or injury; loss of a job; or another life event that they found very stressful. The pilot version of the baseline questionnaire did not include these questions and therefore the pilot subjects (n = 5387) were excluded from analyses of adverse life events. For analyses of age at death of parents, dates of death of parents were obtained from information in the questionnaire about first-degree relatives and were used to compute the subject’s age at bereavement.

### Statistical analysis

Relative risks (RR) with 95 % confidence intervals (CI) for breast cancer were obtained as hazard ratios using Cox proportional hazards models fitted in Stata 14.1 [20] with attained age as the underlying time variable, invasive or in situ breast cancer diagnosis as the event of interest, and censoring at diagnosis, death or end of follow-up, whichever was earliest. Results were adjusted for known breast cancer risk factors that were associated with the stress variables analysed in our data set: age at menarche, age at first birth and parity, cumulative duration of breast feeding, body mass index (BMI) at age 20, postmenopausal BMI and time-updated menopausal status, adult height, oral contraceptive use and postmenopausal combined (oestrogen and progestogen) hormone use, history of benign breast disease, physical activity (in metabolic equivalents, METs, hours/week), alcohol consumption, cigarette smoking, family history of breast cancer and socio-economic status. The latter was based on place of residence (Acorn scores [21]). We also examined hazard ratios adjusted for age only, to assess the likelihood that the results were ‘overmatched’ on lifestyle factors. Results for the two models were similar for most risk factors. Therefore, multivariate-adjusted relative risks are reported throughout and age-adjusted relative risks only where they were materially different. All reported *P* values are two-sided.

We repeated the analyses for premenopausal and postmenopausal women separately, and by oestrogen-receptor status and extent (invasive/in situ) of breast cancer.

## Results

During 2003–2012 a recruitment questionnaire was completed by 106,612 women without a history of breast cancer (Table 1). Follow-up was through to the scheduled completed follow-up questionnaire for 94.6 % of these women. Of the remainder, 1.7 % had developed breast cancer, 0.7 % had died by this time, 2.6 % were alive and had not completed the questionnaire but their vital and cancer status was available from NHSCR, and 0.4 % were lost to follow-up at an earlier date or no follow-up was available. The follow-up rate (calculated as the total observed person-years divided by the maximum person-years that would theoretically have been achievable if there were no loss to follow-up (except breast cancer and deaths)) was 99.5 %, over an average follow-up period of 6.1 years. There were 1783 women who had developed a first invasive (n = 1510) or in situ breast cancer (n = 273), for whom oestrogen-receptor status was known for 92.9 % (99.3 % of invasive, 57.5 % of in situ).

**Table 1:** Baseline characteristics of study participants in the Breakthrough Generations Study recruited 2003–2012

Characteristic	Mean or No.	SD or %
Age at recruitment, years	46.6	13.5
Average person-time, years	6.1	1.0
Menopausal status		
Postmenopausal	41,735	39.1
Premenopausal	59,627	55.9
Status not known	5216	5.0
Never had periods	34	0.0
First-degree family history of breast cancer		
No	90,061	84.5
Yes	16,551	15.5
Age at first full-term pregnancy, years		
≤24	25,982	24.4
25 to 34	46,690	43.8
≥35	4365	4.1
Parous, unknown age	166	0.2
Nulliparous	29,244	27.4
Parity not stated	165	0.2
Ethnicity		
White	105,313	98.8
Other	1299	1.2
Socio-economic status based on place of residence		
1 (highest)	48,632	45.6
2	12,200	11.4
3	30,327	28.4
4	8522	8.0
5 (lowest)	6109	5.7
No classification^a^	822	0.8
Marital status		
Single	13,102	12.3
Married/cohabiting	81,282	76.3
Separated/divorced	8098	7.6
Widowed	3631	3.4
Other or not known	499	0.4
Total	106,612	100.0

^a^507 because they were resident in the Channel Islands, 315 for other reasons

Thirty-four percent of women reported frequent or continuous stress and 74 % at least one adverse life event over the preceding 5 years, ranging from 2.5 % for widowhood to 46.7 % for ‘other life event that they found very stressful’. Women reporting frequent or continuous stress were more likely to have had an adverse life event. Regarding bereavement of parents, 2.5 % of women had lost their mother and 5.1 % their father when they were under 20 years of age. Each of these stress variables was associated with risk factors for breast cancer (Additional file 1: Tables S1–S3).

Relative risks for breast cancer overall in relation to frequency of stress or adverse life events during the 5 years preceding entry to the study ranged between 0.9 and 1.2 and were not statistically significant with the exception of an inverse association with death of a close relative other than a spouse or partner (RR = 0.87, 95 % confidence interval (CI): 0.78–0.97) (Table 2). Relative risks for oestrogen-receptor-positive breast cancer were similar to those for all breast cancers combined. For oestrogen-receptor-negative breast cancer, risk was borderline significantly raised after divorce or separation (RR = 1.54, 95 % CI: 1.01–2.34) and non-significantly reduced after death of a close relative (RR = 0.76, 95 % CI: 0.57–1.02), with analyses for other factors not showing associations.

**Table 2:** Relative risk of breast cancer in relation to experience of stress and adverse life events during the 5 years preceding entry to the study, Breakthrough Generations Study

			Oestrogen-receptor status of breast cancer			
	All cases		Positive		Negative	
Factor	No. of cases	Adjusted RR (95 % CI)^b^	No. of cases	Adjusted RR (95 % CI)^b^	No. of cases	Adjusted RR (95 % CI)^b^
*Frequency of experience of stress* ^*a*^						
Never/occasionally	1112	1.00	871	1.00	161	1.00
Frequently	460	0.92 (0.83–1.03)	355	0.91 (0.81–1.04)	74	1.01 (0.77–1.34)
Continuously	86	0.92 (0.73–1.15)	70	0.96 (0.75–1.23)	9	0.70 (0.36–1.38)
		*P* trend = 0.15		*P* trend = 0.26		*P* trend = 0.55
*Adverse life events* ^*b*^ *:*						
Death of husband/partner						
No	1606	1.00	1255	1.00	236	1.00
Yes	64	1.13 (0.88–1.46)	48	1.07 (0.80–1.44)	11	1.52 (0.82–2.82)
Death of child/parent or other close relative						
No	1200	1.00	933	1.00	185	1.00
Yes	470	0.87 (0.78–0.97)	370	0.88 (0.78–0.99)	62	0.76 (0.57–1.02)
Death of close friend						
No	1390	1.00	1088	1.00	201	1.00
Yes	280	0.94 (0.83–1.08)	215	0.91 (0.79–1.05)	46	1.16 (0.84–1.61)
Personal illness/injury						
No	1520	1.00	1189	1.00	221	1.00
Yes	150	1.03 (0.87–1.22)	114	0.99 (0.82–1.21)	26	1.28 (0.85–1.93)
Loss of job						
No	1534	1.00	1196	1.00	230	1.00
Yes	136	1.09 (0.91–1.30)	107	1.09 (0.89–1.33)	17	0.94 (0.57–1.54)
Divorce/separation						
No	1541	1.00	1207	1.00	222	1.00
Yes	129	1.15 (0.96–1.38)	96	1.09 (0.89–1.33)	25	1.54 (1.01–2.34)
Other stressful life event						
No	929	1.00	745	1.00	124	1.00
Yes	741	0.95 (0.86–1.05)	558	0.90 (0.80–1.00)	123	1.17 (0.91–1.51)
Any of the above						
No	425	1.00	339	1.00	57	1.00
Yes	1245	0.95 (0.85–1.06)	964	0.92 (0.81–1.04)	190	1.12 (0.83–1.52)
Number of events						
0	425	1.00	339	1.00	57	1.00
1	714	0.97 (0.86–1.09)	560	0.95 (0.83–1.09)	107	1.10 (0.80–1.53)
2	373	0.93 (0.81–1.07)	285	0.88 (0.75–1.03)	54	1.07 (0.73–1.55)
≥3	158	0.93 (0.77–1.12)	119	0.87 (0.70–1.07)	29	1.37 (0.87–2.16)
		*P* trend = 0.25		*P* trend = 0.050		*P* trend = 0.25

*RR* relative risk, *CI* confidence interval

^a^Based on 101,225 subjects

^b^Analyses adjusted for attained age, age at menarche, age at first birth and parity, cumulative duration of breast feeding, oral contraceptive use, postmenopausal hormone use, benign breast disease, BMI at age 20, postmenopausal BMI and time-updated menopausal status, height, physical activity, alcohol consumption, cigarette smoking, family history of breast cancer and socio-economic status

Breast cancer risk was not associated with death of a mother or father overall but was significantly raised (age-adjusted RR = 1.55, 95 % CI: 1.22–1.96; multivariate-adjusted RR = 1.31, 95 % CI: 1.02–1.67) in women who were aged less than 20 years at the time of loss of their mother, but not their father (Table 3). In analyses restricted to the 92,441 women whose mother had not had breast or ovarian cancer, this association was attenuated (RR = 1.17, 95 % CI: 0.85–1.61). Relative risks were higher for oestrogen-receptor-negative breast cancer, based on 13 cases, than for oestrogen-receptor-positive breast cancer.

In secondary analyses, results of stress experience for invasive and in situ (n = 1510 and n = 273) and premenopausal and postmenopausal breast cancer separately (n = 1248 and n = 535, respectively) were similar, except for frequent stress, for which a reduced breast cancer risk was observed among postmenopausal (RR = 0.82, 95 % CI: 0.72–0.94) but not premenopausal (RR = 1.18, 95 % CI: 0.97–1.42) women. No association was observed, however, with continuous stress (RR = 0.96, 95 % CI: 0.74–1.25 for postmenopausal and RR = 0.78, 95 % CI: 0.50–1.24 for premenopausal women). With respect to divorce, relative risks were greater for premenopausal (RR = 1.26, 95 % CI: 0.96–1.65) than for postmenopausal (1.04, 95 % CI: 0.81–1.34) women, but neither were statistically significant.

Risk in women who were aged 20 and under at loss of their mother was greater among premenopausal (RR = 1.63, 95 % CI: 1.06–2.51 attenuated to 1.36, 95 % CI: 0.67–2.75 after excluding mothers with breast or ovarian cancer) than postmenopausal (RR = 1.18, 95 % CI: 0.88–1.59 and RR = 1.13, 95 % CI: 0.79–1.62, respectively) women. Relative risks for all mothers were greater for in situ (HR = 1.68, 95 % CI: 0.98–2.90) than invasive (RR = 1.24, 95 % CI: 0.94–1.63) breast cancer. The association for in situ breast cancer was not attenuated after excluding mothers with breast or ovarian cancer (RR = 1.88, 95 % CI: 0.97–3.64), based on ten cases.

## Discussion

Our study has the strengths that it is of prospective design, has a large number of cases with highly complete cohort follow-up, includes a wide range of stress variables and has information enabling analyses by oestrogen-receptor status. In addition, we were able to investigate the effect of adjustment for breast cancer risk factors on relative risk estimates, which is advantageous because the stress variables were associated with behavioural factors such as exercise, smoking, alcohol consumption and obesity.

Women who reported frequent or continuous stress had a comparable risk of breast cancer to those who never or occasionally experienced stress. Our study was fourfold larger than previous studies on this topic, with the exception of the Nurses’ Health Study of care-giving stress, which had a similar number of cases and reported a protective effect for some but not all measures of stress [11]. A lack of association is consistent with the results in two other cohorts [22, 23], but not with three others: one reporting a significant protective effect of self-reported stress [24], and two increased risks in women with raised stress levels [25, 26]. Reasons for these disparities are unclear, but methodological differences in the definition of stress might have contributed. In a recent study of women followed through two menstrual cycles, those who reported high daily stress experience had lower oestradiol levels than those who reported no or little stress [13]. Our study, however, did not observe an inverse association of breast cancer risk with perceived stress in premenopausal women.

Breast cancer risk in our study was not associated with having had any adverse life event during the 5 years preceding entry to the study, or with the total number of such events, a finding supported by previous investigations by the Women’s Health Initiative [27] and the European Prospective Investigation into Cancer [23], but contrasting with several case-control studies [28–31]. We observed, however, a protective effect in those who had lost a close relative other than a spouse/partner, a finding which was weakly supported by that of death of a close friend, but not by the null association with respect to death of a spouse/partner. In the Holmes-Rahe Social Readjustment Rating Scale [14], death of a spouse and divorce receive the highest scores, with death of a close family member being rated fifth. Therefore, given the absence of an association with death of a spouse, the finding for loss of a close relative might be a chance finding. A lack of association with loss of a relative would be consistent with Scandinavian record-linkage studies [32–36], but not with a Finnish prospective study of 10,800 women showing increased risks of breast cancer after death of a spouse, a close relative or friend [37], or with a case-control study showing an increased risk after loss of a close relative [31]. Explanations for the heterogeneity in these findings are unclear but potentially include chance, lack of adjustment for confounders and differences in the timing of the stressor.

**Table 3:** Relative risk of breast cancer in relation to participant’s age at death of parent, Breakthrough Generations Study

			Oestrogen receptor status of breast cancer			
	All cases		Positive		Negative	
Factor	No. of cases	Adjusted RR (95% CI)(a)	No. of Cases	Adjusted RR (95% CI)(a)	No. of cases	Adjusted RR (95% CI)(a)
All mothers						
Mother deceased						
No	916	1.00	700	1.00	146	1.00
Yes	867	0.99 (0.89-1.10)	690	1.00 (0.89-1.13)	121	0.96 (0.72-1.27)
Age of participant at mother’s death, years						
<20	75	1.31 (1.02-1.67)	55	1.21 (0.89-1.61)	13	1.67 (0.93-2.98)
20-29	91	1.02 (0.82-1.27)	72	1.04 (0.81-1.33)	13	1.01 (0.57-1.80)
30-39	178	1.07 (0.91-1.27)	135	1.04 (0.86-1.26)	26	1.09 (0.71-1.68)
40-49	221	0.88 (0.75-1.03)	187	0.95 (0.80-1.13)	23	0.62 (0.39-0.99)
≥50	302	0.94 (0.81-1.10)	241	0.96 (0.81-1.14)	46	1.01 (0.68-1.49)
		P trend=0.059		P trend=0.22		P trend=0.46
Mothers who did not have breast or ovarian cancer						
Mother deceased						
No	790	1.00	603	1.00	128	1.00
Yes	633	0.94 (0.83-1.07)	507	0.98 (0.85-1.13)	88	0.82 (0.60-1.13)
Age of participant at mother’s death, years						
<20	41	1.17 (0.85-1.61)	29	1.05 (0.72-1.55)	7	1.35 (0.62-2.92)
20-29	54	1.04 (0.78-1.38)	43	1.07 (0.78-1.47)	7	0.86 (0.40-1.86)
30-39	119	1.05 (0.86-1.29)	92	1.05 (0.84-1.32)	16	0.92 (0.54-1.57)
40-49	173	0.86 (0.72-1.03)	150	0.98 (0.80-1.19)	16	0.50 (0.28-0.87)
≥50	246	0.89 (0.75-1.05)	193	0.90 (0.75-1.09)	42	0.98 (0.65-1.49)
		P trend=0.046		P trend=0.19		P trend=0.86
All fathers						
Father deceased						
No	730	1.00	568	1.00	104	1.00
Yes	1053	0.95 (0.85-1.06)	822	0.92 (0.81-1.04)	163	1.23 (0.92-1.65)
Age of participant at father’s death, years						
<20	105	0.94 (0.76-1.16)	80	0.88 (0.69-1.12)	18	1.37 (0.82-2.30)
20-29	163	0.96 (0.81-1.15)	133	0.98 (0.80-1.19)	22	1.12 (0.69-1.80)
30-39	259	0.92 (0.79-1.06)	205	0.90 (0.76-1.07)	37	1.08 (0.72-1.61)
40-49	326	1.00 (0.87-1.16)	257	0.98 (0.83-1.15)	49	1.30 (0.89-1.90)
≥50	200	0.91 (0.77-1.09)	147	0.82 (0.67-1.00)	37	1.50 (0.97-2.31)
		P trend=0.97		P trend=0.56		P trend=0.40

*RR* relative risk, *CI* confidence interval

(a) Analyses adjusted for attained age, age at menarche, age at first birth and parity, cumulative duration of breast feeding, oral contraceptive use, postmenopausal hormone use, benign breast disease, BMI at age 20, postmenopausal BMI and time-updated menopausal status, height, physical activity, alcohol consumption, cigarette smoking, family history of breast cancer and socioeconomic status

We did not observe an association with divorce, except for a positive association for oestrogen-receptor-negative breast cancer only. No previous studies reported on associations by receptor status, and given that we are the first to report this association, it should be investigated by other studies. Our results hinted that the association might be restricted to premenopausal women only, which would be consistent as premenopausal women are more likely than postmenopausal women to have oestrogen-receptor-negative breast cancer [38]. A twofold increased risk of breast cancer overall after divorce has been previously reported by a prospective study [37], but a record-linkage study [32] reported a significant 20 % reduced risk of breast cancer among divorced compared with married women, with no or weak associations reported by case-control studies [29, 39].

Bereavement during childhood and adolescence has a well-established long-term impact on both mental and physical health [5, 40–42] and it is possible that it affects subsequent breast cancer risk. A direct role of cortisol on breast tissue development and oestrogen production and activity has been proposed as a potential mechanism [9], but changes in lifestyle subsequent to bereavement and familial factors could also play a role. Breast cancer risk after maternal death at young ages has not previously been investigated except for in two small prospective studies, one showing a 2.6-fold increased risk [43] and the other reporting no association [44]. In our study, having been bereaved under age 20 of a mother, but not of a father, was associated with a significant increase in breast cancer risk. This association was strongest for premenopausal women and for oestrogen-receptor-negative breast cancer. Women whose mother had breast cancer at young ages are at increased risk of having breast cancer at young ages themselves [45], and our risk estimates were attenuated when analyses were restricted to women whose mother did not have breast or ovarian cancer. It appears, therefore, that the observed associations are at least in part due to increased susceptibility to breast cancer through genetic inheritance. The stronger association for in situ than invasive cancer is possibly due to enhanced breast cancer screening among women who lost their mother early, but it is unclear why this association appeared to be somewhat stronger for women whose mother did not have breast or ovarian cancer. This was based on a small number of cases, however.

Limitations of our study are the lack of information on the intensity of stress, on stress in the workplace, and on the extent of social support or stress adaptive capacity, although the latter measure was not associated with breast cancer risk in a previous study [23]. Furthermore, most of the analysed stress variables were collected for the 5 years prior to completion of the baseline questionnaire only; we could not examine effects of such events in earlier time windows. Our study did not collect information on stressful events during childhood or adolescence with the exception of loss of a parent. Future studies should investigate stress and adverse events in such earlier time windows, as well as focus on populations for which our data were sparser, i.e. investigate effects of divorce and other adverse events on breast cancer in young, premenopausal women, by breast cancer characteristics.

## Conclusions

In this large prospective study there was no consistent evidence that self-reported frequency of stress and experience of adverse life events affected subsequent breast cancer risk. Raised risks in women who were bereaved of their mother during childhood or adolescence were at least in part due to familial susceptibility.

## Abbreviations

BMI, body mass index; CI, confidence interval; MET, metabolic equivalent; NHSCR, National Health Service Central Registers; RR, relative risk; UK, United Kingdom

## Additional file

Additional file 1: Association of stress variables with breast cancer risk factors. **Table S1.** Association of frequency of experience of stress during the 5 years preceding entry to the study with breast cancer risk factors and other stress variables assessed at recruitment. **Table S2.** Association of experience of adverse life events during the 5 years preceding entry to the study with breast cancer risk factors and other stress variables assessed at recruitment. **Table S3.** Association of participant’s age at death of their mother with breast cancer risk factors and other stress variables assessed at recruitment. (DOC 104 kb)
