# Supplementary material for: Psychological stress, adverse life events and breast cancer incidence: a cohort investigation in 106,000 women in the United Kingdom
Source: Breast Cancer Res. 2016 Jul 15;18:72. doi: 10.1186/s13058-016-0733-1 (PMC4946095; doi:10.1186/s13058-016-0733-1)
Supplement: Additional file 1: — Association of stress variables with breast cancer risk factors. Table S1. Association of frequency of experience of stress during the 5 years preceding entry to the study with breast cancer risk factors and other stress variables assessed at recruitment. Table S2. Association of experience of adverse life events during the 5 years preceding entry to the study with breast cancer risk factors and other stress variables assessed at recruitment. Table S3. Association of participant’s age at death of their mother with breast cancer risk factors and other stress variables assessed at recruitment. (DOC 104 kb) [file 13058_2016_733_MOESM1_ESM.doc]

Additional file 1

Table S1. Association of frequency of experience of stress during the five years preceding entry to the study with breast cancer risk factors and other stress variables assessed at recruitment

|  | Frequency of experience of stress during the  5 years preceding study entry | | | | | | | | |
| --- | --- | --- | --- | --- | --- | --- | --- | --- | --- |
| Factor, at entry to the study | Never/  occasionally | | Frequently | Con-tinuously | | | | | P-trend |
| Mean age at entry to study, years | 46.8 | | 46.2 | 47.2 | | | | |  |
|  |  | | *Age-adjusted means* | |  | | | |  |
| Age at menarche, years | 12.7 | | 12.6 | 12.6 | | | | | <0.001 |
| Age at first birth, years (a) | 26.7 | | 26.6 | 25.9 | | | | | <0.001 |
| Parity (a) | 2.17 | | 2.18 | 2.21 | | | | | 0.009 |
| Breast feeding, cumulative weeks (a) | 42.3 | | 43.9 | 40.3 | | | | | 0.33 |
| Body mass index at age 20, kg/m2 | 21.7 | | 21.8 | 21.9 | | | | | 0.005 |
| Body mass index at entry, kg/m2 | 25.1 | | 25.6 | 26.5 | | | | | <0.001 |
| Adult height, cms | 164.4 | | 164.3 | 163.9 | | | | | <0.001 |
| Physical activity, MET hours/week | 62.7 | | 64.4 | 68.6 | | | | | <0.001 |
| Alcoholic units, per week | 12.6 | | 13.2 | 13.8 | | | | | <0.001 |
|  |  | |  |  | | | | |  |
|  |  | *Age-adjusted percentages* | | | |  | |  | |
| % parous | 73.2 | | 74.9 | 77.9 | | | <0.001 | | |
| % current cigarette smokers | 6.2 | | 8.7 | 12.0 | | | <0.001 | | |
| % benign breast disease | 17.9 | | 22.4 | 23.3 | | | <0.001 | | |
| % family history of breast cancer | 14.7 | | 16.0 | 17.3 | | | <0.001 | | |
| % postmenopausal | 41.6 | | 41.1 | 42.2 | | | 0.46 | | |
| % current oral contraception use (b) | 26.2 | | 23.6 | 20.7 | | | <0.001 | | |
| % current postmenopausal hormone use (c) | 6.7 | | 7.1 | 7.6 | | | 0.029 | | |
| % high socioeconomic status | 47.6 | | 44.4 | 41.1 | | | <0.001 | | |
| % with adverse life event, past 5 years | 66.7 | | 87.1 | 94.2 | | | <0.001 | | |
| % who lost mother under age 20 | 2.5 | | 2.5 | 2.4 | | | 0.84 | | |
|  |  | |  |  | | |  | | |

MET: Metabolic Equivalent

1. Among parous women only
2. Among premenopausal women only
3. Among postmenopausal women only

Table S2. Association of experience of adverse life events during the five years preceding entry to the study with breast cancer risk factors and other stress variables assessed at recruitment

|  | | | Adverse life event during  5 years preceding study entry | | | | | |
| --- | --- | --- | --- | --- | --- | --- | --- | --- |
| Factor, at entry to the study | | | None | 1 event | >1 event | | P-trend | |
| Mean age at entry to study, years | | | 45.5 | 46.6 | 47.6 | |  | |
|  | | |  | *Age-adjusted means* | |  |  | |
| Age at menarche, years | | | 12.8 | 12.7 | 12.6 | | <0.001 | |
| Age at first birth, years (a) | | | 27.2 | 26.6 | 26.2 | | <0.001 | |
| Parity (a) | | | 2.22 | 2.18 | 2.15 | | <0.001 | |
| Breast feeding, cumulative weeks (a) | | | 47.0 | 41.7 | 40.1 | | <0.001 | |
| Body mass index at age 20, kg/m2 | | | 21.6 | 21.7 | 21.9 | | <0.001 | |
| Body mass index at entry, kg/m2 | | | 24.8 | 25.2 | 25.8 | | <0.001 | |
| Adult height, cms | | | 164.5 | 164.3 | 164.3 | | <0.001 | |
| Physical activity, MET hours/week | | | 58.2 | 63.2 | 68.2 | | <0.001 | |
| Alcoholic units, per week | | | 12.9 | 12.6 | 13.1 | | 0.013 | |
|  | | |  |  |  | |  | |
|  | | |  | *Age-adjusted percentages* | | |  |  |
| % parous | | | 74.5 | 74.7 | 72.7 | | <0.001 | |
| % current cigarette smokers | | | 4.9 | 7.1 | 9.4 | | <0.001 | |
| % benign breast disease | | | 17.1 | 19.5 | 21.5 | | <0.001 | |
| % family history of breast cancer | | | 13.3 | 15.2 | 16.6 | | <0.001 | |
| % postmenopausal | | | 40.0 | 41.3 | 43.2 | | <0.001 | |
| % current oral contraceptive use (b) | | | 25.9 | 25.1 | 24.5 | | 0.001 | |
| % current postmenopausal hormone use (c) | | | 7.1 | 6.7 | 6.9 | | 0.50 | |
| % high socioeconomic status | 50.0 | | | 46.7 | 42.7 | | <0.001 | |
| % reporting frequent/continuous stress | 15.5 | | | 33.1 | 50.9 | | <0.001 | |
| % who lost mother under age 20 | 2.6 | | | 2.4 | 2.5 | | 0.21 | |
|  | |  | |  |  | |  | |

MET: Metabolic Equivalent

1. Among parous women only
2. Among premenopausal women only
3. Among postmenopausal women only

Table S3. Association of participant’s age at death of their mother with breast cancer risk factors and other stress variables assessed at recruitment

|  | Participant’s age at mother’s death, years | | | | | |
| --- | --- | --- | --- | --- | --- | --- |
| Factor, at entry to the study | <20 | | ≥20 | Mother alive | | P-heterogeneity |
| Age at entry to study, years | 49.7 | | 57.3 | 41.4 | |  |
|  |  | | *Age-adjusted means* | |  |  |
| Age at menarche, years | 12.7 | | 12.6 | 12.7 | | <0.001 |
| Age at first birth, years (a) | 26.2 | | 26.4 | 26.7 | | <0.001 |
| Parity (a) | 2.19 | | 2.16 | 2.19 | | <0.001 |
| Breast feeding, cumulative weeks (a) | 40.2 | | 39.6 | 44.1 | | <0.001 |
| Body mass index at age 20, kg/m2 | 21.9 | | 21.9 | 21.7 | | <0.001 |
| Body mass index at entry, kg/m2 | 25.5 | | 25.4 | 25.3 | | 0.002 |
| Adult height, cms | 163.9 | | 164.3 | 164.4 | | <0.001 |
| Physical activity, MET hours/week | 65.4 | | 65.2 | 62.8 | | <0.001 |
| Alcoholic units, per week | 12.8 | | 12.7 | 13.0 | | 0.008 |
|  |  | |  |  | |  |
|  |  | *Age-adjusted percentages* | | |  |  |
| % parous | 70.9 | | 69.3 | 76.0 | | <0.001 |
| % current cigarette smokers | 7.8 | | 7.7 | 7.2 | | 0.019 |
| % benign breast disease | 21.5 | | 19.6 | 19.4 | | 0.021 |
| % family history of breast cancer | 38.2 | | 24.5 | 10.5 | | <0.001 |
| % postmenopausal | 41.8 | | 46.8 | 38.5 | | <0.001 |
| % current oral contraceptive use (b) | 23.6 | | 26.3 | 25.1 | | 0.008 |
| % current postmenopausal hormone use (c) | 7.3 | | 7.1 | 7.0 | | 0.89 |
| % high socioeconomic status | 41.7 | | 44.6 | 46.8 | | <0.001 |
| % reporting frequent/continuous stress | 34.1 | | 33.6 | 34.3 | | 0.21 |
| % adverse life event, past 5 years | 72.9 | | 79.3 | 71.3 | | <0.001 |
|  |  | |  |  | |  |

MET: Metabolic Equivalent

1. Among parous women only
2. Among premenopausal women only
3. Among postmenopausal women only
